# Supplementary material for: Factors influencing open government data post-adoption in the public sector: The perspective of data providers
Source: PLoS One. 2022 Nov 2;17(11):e0276860. doi: 10.1371/journal.pone.0276860 (PMC9629594; doi:10.1371/journal.pone.0276860)
Supplement: S1 Table — (DOCX) [file pone.0276860.s001.docx]

S1 Appendix. The Results for Measurement Model Assessment.

**S1A Table. The Result for Cross Loadings.**

|  | **ACC** | **CPB** | **CPX** | **OGC** | **DAD** | **ITC** | **INC** | **INF** | **RAD** | **RTN** | **TMS** |
| --- | --- | --- | --- | --- | --- | --- | --- | --- | --- | --- | --- |
| **ACC1** | 0.705 | 0.354 | −0.406 | 0.298 | 0.187 | 0.262 | 0.267 | 0.382 | 0.218 | 0.462 | 0.329 |
| **ACC2** | 0.866 | 0.378 | −0.323 | 0.429 | 0.4 | 0.263 | 0.257 | 0.549 | 0.352 | 0.558 | 0.53 |
| **ACC3** | 0.869 | 0.399 | −0.234 | 0.417 | 0.338 | 0.358 | 0.223 | 0.597 | 0.461 | 0.603 | 0.459 |
| **ACC4** | 0.805 | 0.419 | −0.169 | 0.496 | 0.445 | 0.204 | 0.281 | 0.574 | 0.442 | 0.502 | 0.497 |
| **CPB1** | 0.395 | 0.85 | −0.313 | 0.464 | 0.409 | 0.255 | 0.329 | 0.522 | 0.527 | 0.42 | 0.415 |
| **CPB2** | 0.444 | 0.889 | −0.291 | 0.515 | 0.457 | 0.322 | 0.321 | 0.562 | 0.594 | 0.483 | 0.428 |
| **CPB3** | 0.336 | 0.772 | −0.378 | 0.376 | 0.286 | 0.334 | 0.332 | 0.396 | 0.395 | 0.369 | 0.371 |
| **CPB4** | 0.395 | 0.803 | −0.098 | 0.48 | 0.498 | 0.219 | 0.234 | 0.542 | 0.673 | 0.467 | 0.371 |
| **CPX1** | −0.296 | −0.308 | 0.829 | −0.226 | −0.245 | −0.193 | −0.156 | −0.208 | −0.098 | −0.223 | −0.2 |
| **CPX2** | −0.291 | −0.278 | 0.812 | −0.201 | −0.186 | −0.203 | −0.144 | −0.247 | −0.101 | −0.248 | −0.229 |
| **CPX3** | −0.301 | −0.261 | 0.857 | −0.143 | −0.176 | −0.228 | −0.14 | −0.214 | −0.092 | −0.29 | −0.239 |
| **CPX4** | −0.169 | −0.148 | 0.714 | −0.039 | −0.06 | −0.231 | −0.033 | −0.086 | −0.005 | −0.161 | −0.059 |
| **OGC1** | 0.44 | 0.526 | −0.205 | 0.869 | 0.366 | 0.362 | 0.249 | 0.495 | 0.434 | 0.442 | 0.449 |
| **OGC2** | 0.408 | 0.463 | −0.152 | 0.857 | 0.428 | 0.252 | 0.21 | 0.467 | 0.406 | 0.355 | 0.511 |
| **OGC3** | 0.465 | 0.456 | −0.169 | 0.867 | 0.393 | 0.266 | 0.297 | 0.486 | 0.377 | 0.406 | 0.557 |
| **DAD1** | 0.407 | 0.525 | −0.222 | 0.458 | 0.901 | 0.084 | 0.33 | 0.508 | 0.433 | 0.353 | 0.391 |
| **DAD2** | 0.366 | 0.463 | −0.213 | 0.401 | 0.904 | 0.104 | 0.321 | 0.434 | 0.405 | 0.297 | 0.333 |
| **DAD4** | 0.325 | 0.285 | −0.124 | 0.305 | 0.764 | 0.197 | 0.163 | 0.388 | 0.333 | 0.404 | 0.318 |
| **INC1** | 0.299 | 0.281 | −0.138 | 0.234 | 0.246 | 0.282 | 0.766 | 0.291 | 0.282 | 0.322 | 0.391 |
| **INC2** | 0.179 | 0.319 | −0.069 | 0.184 | 0.291 | 0.01 | 0.754 | 0.212 | 0.231 | 0.077 | 0.223 |
| **INC3** | 0.251 | 0.252 | −0.094 | 0.261 | 0.208 | 0.191 | 0.806 | 0.286 | 0.194 | 0.245 | 0.276 |
| **INC4** | 0.179 | 0.268 | −0.179 | 0.195 | 0.252 | 0.068 | 0.703 | 0.21 | 0.166 | 0.118 | 0.167 |
| **INF1** | 0.535 | 0.472 | −0.278 | 0.479 | 0.391 | 0.344 | 0.349 | 0.741 | 0.387 | 0.546 | 0.549 |
| **INF2** | 0.572 | 0.543 | −0.197 | 0.458 | 0.437 | 0.288 | 0.279 | 0.889 | 0.529 | 0.661 | 0.485 |
| **INF3** | 0.494 | 0.492 | −0.234 | 0.416 | 0.388 | 0.328 | 0.254 | 0.834 | 0.462 | 0.549 | 0.426 |
| **INF4** | 0.556 | 0.523 | −0.117 | 0.499 | 0.503 | 0.199 | 0.244 | 0.84 | 0.548 | 0.586 | 0.418 |
| **ITC1** | 0.336 | 0.3 | −0.191 | 0.348 | 0.092 | 0.853 | 0.191 | 0.343 | 0.23 | 0.387 | 0.371 |
| **ITC2** | 0.173 | 0.137 | −0.264 | 0.129 | -0.046 | 0.72 | 0.049 | 0.103 | 0.086 | 0.336 | 0.208 |
| **ITC3** | 0.3 | 0.364 | −0.192 | 0.346 | 0.233 | 0.83 | 0.233 | 0.357 | 0.322 | 0.381 | 0.347 |
| **ITC4** | 0.175 | 0.19 | −0.214 | 0.142 | 0.117 | 0.729 | 0.136 | 0.193 | 0.157 | 0.23 | 0.201 |
| **RAD1** | 0.382 | 0.558 | −0.007 | 0.404 | 0.341 | 0.195 | 0.266 | 0.464 | 0.819 | 0.414 | 0.327 |
| **RAD2** | 0.444 | 0.612 | −0.185 | 0.432 | 0.444 | 0.256 | 0.304 | 0.541 | 0.879 | 0.465 | 0.407 |
| **RAD3** | 0.416 | 0.586 | −0.108 | 0.415 | 0.419 | 0.257 | 0.247 | 0.529 | 0.879 | 0.456 | 0.39 |
| **RAD4** | 0.237 | 0.418 | 0.017 | 0.268 | 0.28 | 0.192 | 0.113 | 0.377 | 0.733 | 0.381 | 0.242 |
| **ROU1** | 0.524 | 0.479 | −0.165 | 0.402 | 0.385 | 0.242 | 0.177 | 0.608 | 0.461 | 0.779 | 0.448 |
| **ROU2** | 0.613 | 0.509 | −0.334 | 0.426 | 0.321 | 0.44 | 0.281 | 0.593 | 0.455 | 0.889 | 0.552 |
| **ROU3** | 0.536 | 0.429 | −0.284 | 0.361 | 0.291 | 0.435 | 0.217 | 0.59 | 0.39 | 0.846 | 0.526 |
| **ROU4** | 0.481 | 0.307 | −0.177 | 0.34 | 0.344 | 0.311 | 0.243 | 0.552 | 0.402 | 0.78 | 0.427 |
| **TMS1** | 0.469 | 0.387 | −0.193 | 0.51 | 0.312 | 0.336 | 0.319 | 0.423 | 0.293 | 0.47 | 0.794 |
| **TMS2** | 0.453 | 0.417 | −0.261 | 0.518 | 0.338 | 0.4 | 0.286 | 0.454 | 0.355 | 0.535 | 0.872 |
| **TMS3** | 0.433 | 0.37 | −0.207 | 0.391 | 0.282 | 0.349 | 0.344 | 0.435 | 0.341 | 0.475 | 0.822 |
| **TMS4** | 0.487 | 0.391 | −0.137 | 0.493 | 0.393 | 0.173 | 0.266 | 0.536 | 0.393 | 0.467 | 0.79 |

ACC: Acceptance, CPB: Compatibility, CPX: Complexity, OGC: Organizational Culture, Data Demand: DAD, ITC: IT Competency, INC: Incentives, INF: Infusion, RAD: Relative advantage, RTN: Routinization, TMS: Top management support

**S1B Table. The Result for Fornell–Larcker’s Assessment.**

|  | **ACC** | **CPB** | **CPX** | **OGC** | **DAD** | **ITC** | **INC** | **INF** | **RAD** | **RTN** | **TMS** |
| --- | --- | --- | --- | --- | --- | --- | --- | --- | --- | --- | --- |
| **ACC** | 0.814 |  |  |  |  |  |  |  |  |  |  |
| **CPB** | 0.476 | 0.83 |  |  |  |  |  |  |  |  |  |
| **CPX** | −0.339 | −0.32 | 0.805 |  |  |  |  |  |  |  |  |
| **OGC** | 0.508 | 0.557 | −0.203 | 0.864 |  |  |  |  |  |  |  |
| **DAD** | 0.428 | 0.503 | −0.22 | 0.457 | 0.859 |  |  |  |  |  |  |
| **ITC** | 0.335 | 0.339 | −0.26 | 0.34 | 0.144 | 0.785 |  |  |  |  |  |
| **INC** | 0.313 | 0.365 | −0.158 | 0.294 | 0.322 | 0.211 | 0.758 |  |  |  |  |
| **INF** | 0.653 | 0.614 | −0.247 | 0.559 | 0.52 | 0.347 | 0.338 | 0.828 |  |  |  |
| **RAD** | 0.46 | 0.666 | −0.101 | 0.468 | 0.457 | 0.274 | 0.295 | 0.585 | 0.83 |  |  |
| **RTN** | 0.656 | 0.527 | −0.294 | 0.465 | 0.406 | 0.435 | 0.278 | 0.711 | 0.519 | 0.825 |  |
| **TMS** | 0.563 | 0.478 | −0.242 | 0.586 | 0.406 | 0.381 | 0.37 | 0.566 | 0.423 | 0.594 | 0.82 |

ACC: Acceptance, CPB: Compatibility, CPX: Complexity, OGC: Organizational Culture, Data Demand: DAD, ITC: IT Competency, INC: Incentives, INF: Infusion, RAD: Relative advantage, RTN: Routinization, TMS: Top management support

**S1C Table. The Result for HTMT.**

|  | **ACC** | **CPB** | **CPX** | **OGC** | **DAD** | **ITC** | **INC** | **INF** | **RAD** | **RTN** | **TMS** |
| --- | --- | --- | --- | --- | --- | --- | --- | --- | --- | --- | --- |
| **ACC** |  |  |  |  |  |  |  |  |  |  |  |
| **CPB** | 0.567 |  |  |  |  |  |  |  |  |  |  |
| **CPX** | 0.41 | 0.379 |  |  |  |  |  |  |  |  |  |
| **OGC** | 0.607 | 0.659 | 0.234 |  |  |  |  |  |  |  |  |
| **DAD** | 0.51 | 0.587 | 0.248 | 0.549 |  |  |  |  |  |  |  |
| **ITC** | 0.383 | 0.384 | 0.343 | 0.372 | 0.222 |  |  |  |  |  |  |
| **INC** | 0.381 | 0.46 | 0.192 | 0.358 | 0.405 | 0.273 |  |  |  |  |  |
| **INF** | 0.775 | 0.72 | 0.289 | 0.668 | 0.622 | 0.408 | 0.411 |  |  |  |  |
| **RAD** | 0.521 | 0.762 | 0.122 | 0.544 | 0.533 | 0.3 | 0.335 | 0.676 |  |  |  |
| **RTN** | 0.782 | 0.616 | 0.342 | 0.554 | 0.495 | 0.51 | 0.319 | 0.841 | 0.61 |  |  |
| **TMS** | 0.67 | 0.566 | 0.28 | 0.698 | 0.486 | 0.45 | 0.435 | 0.673 | 0.485 | 0.706 |  |

ACC: Acceptance, CPB: Compatibility, CPX: Complexity, OGC: Organizational Culture, Data Demand: DAD, ITC: IT Competency, INC: Incentives, INF: Infusion, RAD: Relative advantage, RTN: Routinization, TMS: Top management support
