# Supplementary material for: Factors influencing open government data post-adoption in the public sector: The perspective of data providers
Source: PLoS One. 2022 Nov 2;17(11):e0276860. doi: 10.1371/journal.pone.0276860 (PMC9629594; doi:10.1371/journal.pone.0276860)
Supplement: S1 Appendix — (DOCX) [file pone.0276860.s002.docx]

**S1 Appendix. Survey Questionnaire for Open Government Data Post-Adoption Factors Among Data Providers.**

**Section A: Demographic**

Multiple choice

1. What is your present position in your organization?

*(Executive Level Management/ Middle-Level management/ Lower-Level Management/ Operational Level)*

1. What is your job title?
2. Gender *(Male/Female)*
3. Age *(60 years old and above/ 50 - 59 years old/ 40 - 49 years old/ 30 - 39 years old/ 21 - 29 years old/ 20 years and below)*
4. How long have you served in government?

*(More than 30 years/ Between 21 to 30 years/ Between 11 to 20 years/ Between 5 to 10 years/ Less than 5 years)*

1. How long have you been involved in Open Government Data initiatives?

*(More than 10 years/ Between 5 to less than 10 years/ Between 2 to less than 5 years/ Less than 2 years)*

1. Which of the following does your organization/agency belong to?

*(Federal/Federal Statutory Body/Local Authority/ State/State Statutory Body/ Corporation/ Others)*

1. How many IT personnel are there in your organization/agency?

*(More than 50 persons/ Between 31 to 50 persons/ Between 11 to 30 persons/ Between 5 to 10 persons/ Between 1 to 4 persons/ None)*

**Section B: Open Government Data Post-Adoption Factors**

Likert-scale

| Strongly Disagree | Disagree | Neutral | Agree | Strongly Agree |
| --- | --- | --- | --- | --- |
| 1 | 2 | 3 | 4 | 5 |

**Compatibility**

1. Open Government Data initiatives are compatible with the data captured at our agency.
2. Open Government Data initiatives are suited to our agency’s existing operating practices.
3. Open Government Data initiatives are compatible with our agency’s IT infrastructure.
4. Open Government Data initiative is consistent with our agency’s values and beliefs.

**Complexity**

1. Our agency finds publishing Open Government Data is a complex process.
2. Our agency faces difficulty in categorizing data that can be published as Open Government Data.
3. Our agency finds it is challenging to publish Open Government Data with high granularity.
4. Our agency’s data need to go through a complex process before being published as Open Government Data.

**Relative advantage**

| 1. Open Government Data implementation increases the performance of our agency’s operation. |
| --- |
| 1. Open Government Data implementation raises the efficiency of our agency’s operation. |
| 1. Open Government Data implementation enhances the effectiveness of our agency’s operation. |
| 1. Open Government Data provides our agency with valuable information to make decisions. |

**Top management support**

| 1. Top management in our agency is articulating a vision for Open Government Data implementation. |
| --- |
| 1. Top management in our agency is formulating a strategy for Open Government Data implementation. |
| 1. Top management in our agency is deploying the Open Government Data initiative implementation efforts. |
| 1. Top management in our agency is giving attention to the performance of the Open Government Data initiatives implementation. |

**Organization culture**

| 1. Our agency is willing to share information and data with the public. |
| --- |
| 1. Our agency encourages the practice of information and data sharing with the public. |
| 1. Our agency is open to innovative policies such as sharing information and data with the public. |
| 1. Our agency has implemented the open government data sharing policy. |

**IT Competency**

| 1. Our agency is committed to assuring that the staff is familiar with Open Government Data initiatives. |
| --- |
| 1. Our agency has a sound knowledge of Open Government Data initiatives. |
| 1. Our agency has the technological resources to manage Open Government Data implementation. |
| 1. Our agency’s staff is able to use their experience and knowledge to operate Open Government Data implementation. |

**Data demand**

| 1. Our agency regards the data request from the public as part of the government service to the people. |
| --- |
| 1. Our agency believes that the use of open data from the public will influence our agency to publish Open Government Data. |
| 1. Our agency only accepts data requests for the already available datasets to be published. |
| 1. Our agency finds that fulfilling the demand for Open Government Data by the public is a satisfying task |

**Incentives**

| 1. The superior level agency provides our agency with incentives to implement Open Government Data initiatives. |
| --- |
| 1. An incentive from the superior level agency is essential for the agency at the bottom level to implement Open Government Data initiatives. |
| 1. Our agency is more motivated to implement Open Government Data initiatives if the incentive is provided. |
| 1. There is recognition provided by an external party (non-governmental/private bodies, etc.) for government agencies implementing the Open Government Data initiative. |

**Acceptance**

| 1. Our agency publishes Open Government Data voluntarily. |
| --- |
| 1. Open government data initiative is well accepted in our agency. |
| 1. Our agency is satisfied with the Open Government Data implementation in our agency. |
| 1. Our agency published Open Government Data as frequently as possible. |

**Routinization**

| 1. Our agency publishes Open Government Data on a regular basis. |
| --- |
| 1. Open Government Data publication has become a normal operation in our agency. |
| 1. Open Government Data publication is regarded as a regular activity in our agency. |
| 1. Our agency’s work system is adapted well to Open Government Data initiatives. |

**Infusion**

| 1. The Open Government Data policy has been fully adopted by our agency. |
| --- |
| 1. Our agency has incorporated the publication of Open Government Data into agency work norms. |
| 1. Our agency has implemented the Open Government Data initiatives in accordance with the guidelines set out. |
| 1. Open Government Data publication is an integral part of our agency’s activity. |
