# Supplementary material for: Factors influencing open government data post-adoption in the public sector: The perspective of data providers
Source: PLoS One. 2022 Nov 2;17(11):e0276860. doi: 10.1371/journal.pone.0276860 (PMC9629594; doi:10.1371/journal.pone.0276860)
Supplement: S2 Appendix — (DOCX) [file pone.0276860.s003.docx]

S2 Appendix. Interview scripts for Semi-structured Interviews during Preliminary Study.

| 1. **A. Profile of an interviewee** |
| --- |
| 1. How long have you been working in government services?  2. How long have you been involved in open data initiatives in the Malaysian public sector?  3. What is your role in open data initiatives in Malaysian public sectors? |
| 1. **B. OGD Implementation Landscape** |
| 1. What is the current progress of open government data (OGD) implementation in Malaysian public sectors? 2. How do you see the acceptance of the OGD initiatives amongst government agencies? 3. In your opinion, is it important for the OGD initiatives to be part of the government agency’s core business? 4. In your opinion, what are the essential actions the government agency should take in the post-adoption phase to ensure the continuity of OGD initiatives? 5. Do you think it would be beneficial for the agency if OGD initiatives became part of its basic operations? |
| 1. **C. Post-adoption of OGD Factors** |
| 1. In what way does top-level leadership of the agency facilitate an OGD initiative? 2. Is there a management structure to manage OGD initiatives at the agency level? 3. How important is data governance in the government agency for managing OGD projects? 4. Do government agencies ever refuse to release information that qualifies as OGD? 5. Is there any incentive/reward given to the agency that participated in OGD initiatives? 6. Is it important for central agencies to offer incentives or rewards to encourage agencies to take part in OGD initiatives? 7. Do government agencies find it hard and complicated to provide OGD? 8. Do government agencies consider delivering OGD to be an additional burden on their regular tasks? 9. Do government agencies find OGD compatible with their agency’s vision and mission? 10. Do you think sharing data publicly could lead to any negative consequences to any government agency? Why? 11. Are you satisfied with the current availability of open data in the government open data portal? Is it of high value and quality? 12. Do government agencies have to abide by certain data policies before releasing data to the public? If yes, what is the policy? 13. Do government agencies consider privacy and security risks before providing open data sets? 14. Does opening government data involve a high-tech IT infrastructure? 15. Do government agencies’ current IT infrastructure (e.g., data center, network) sufficient to sustain OGD initiatives in the future? 16. How important is technical IT competency in OGD implementation among government agencies? 17. Do government agencies’ personnel need special IT training in managing OGD initiatives? 18. Do government agencies value requests for data from the general public or other agencies? 19. What other factors could influence government agencies to continue implementing OGD initiatives in the long-term? |
