# Supplementary material for: Factors influencing open government data post-adoption in the public sector: The perspective of data providers
Source: PLoS One. 2022 Nov 2;17(11):e0276860. doi: 10.1371/journal.pone.0276860 (PMC9629594; doi:10.1371/journal.pone.0276860)
Supplement: Attachment — Submitted filename: Response to Reviewer.pdf [file pone.0276860.s005.pdf]

Response to Reviewer

Reviewer #1:

1. "Yet, the underlying factors influencing OGD after the adoption phase are scarce". The factors are scarce OR studies are scarce? Please confirm the intended meaning of the sentence.

Response: Thank you for highlighting this. We have clarified the statement at line 30 as follows.

*"Yet, **studies on** the underlying factors influencing OGD after the adoption phase are scarce".*

2. In response to our previous observation No. 13 i.e. "Line 215, How can you determine that TOE is the most recognized attempt…? Any reference? Strong justification is necessary here.", You may add two references, particularly in the open data context, here (1) Modeling of Open Government Data for Public Sector Organizations Using the Potential Theories and Determinants—A Systematic Review (2) Factors Affecting Performance of Open Government Data Initiatives: A Multi-Method Approach Using Sem and FSQCA.

Response: Thank you for your suggestion. We have added the two references on lines 284-296 to justify the TOE is the most utilized theory in IS/IT adoption study at an organizational level. Apologize for overlooking these highly relevant papers to our study.

Journal Requirements:

There are two new references added to the manuscript as follows:

5. Hossain MA, Rahman S, Quaddus M, Hooi E, Olanrewaju A-S. Factors Affecting Performance of Open Government Data Initiatives: A Multi-Method Approach Using Sem and FSQCA. Journal of Organizational Computing and Electronic Commerce. 2021;31(4):300-19.

72. Khurshid MM, Zakaria NH, Rashid A, Ahmad MN, Arfeen MI, Faisal Shehzad HM, editors. Modeling of open government data for public sector organizations using the potential theories and determinants—a systematic review. Informatics; 2020: MDPI.

CONCLUDING REMARKS:

.

Dear Reviewers,

We have made the adjustment as commented. We truly hope that the revised manuscript is now clarified enough for the reviewer to take the next step. Finally, we would like to express our immense gratitude for your help on improving the readability of our paper.

Sincerely,

Associate Prof. Dr. Suraya Hamid
Corresponding Author
Faculty of Computer Science and Information Technology
Universiti Malaya
50603 Kuala Lumpur
Malaysia
email: suraya_hamid@um.edu.my
